# Coordinate Based Meta-Analysis of Functional Neuroimaging Data Using Activation Likelihood Estimation; Full Width Half Max and Group Comparisons

**DOI:** 10.1371/journal.pone.0106735

**Published:** 2014-09-16

**Authors:** Christopher R. Tench, Radu Tanasescu, Dorothee P. Auer, William J. Cottam, Cris S. Constantinescu

**Affiliations:** 1 Division of Clinical Neurosciences, Clinical Neurology, University of Nottingham, Queen's Medical Centre, Nottingham, United Kingdom; 2 Department of Neurology, Neurosurgery, and Psychiatry, University of Medicine and Pharmacy Carol Davila Bucharest, Colentina Hospital, Bucharest, Romania; 3 Division of Clinical Neurosciences, Radiological and Imaging Sciences, University of Nottingham, Queen's Medical Centre, Nottingham, United Kingdom; 4 ARUK National Pain Centre, University of Nottingham, Queen's Medical Centre, Nottingham, United Kingdom; Wake Forest School of Medicine, United States of America

## Abstract

Coordinate based meta-analysis (CBMA) is used to find regions of consistent activation across fMRI and PET studies selected for their functional relevance to a hypothesis. Results are clusters of foci where multiple studies report in the same spatial region, indicating functional relevance. Contrast meta-analysis finds regions where there are consistent differences in activation pattern between two groups. The activation likelihood estimate methods tackle these problems, but require a specification of uncertainty in foci location: the full width half max (FWHM). Results are sensitive to FWHM. Furthermore, contrast meta-analysis requires correction for multiple statistical tests. Consequently it is sensitive only to very significant localised differences that produce very small p-values, which remain significant after correction; subtle diffuse differences between the groups can be overlooked. In this report we redefine the FWHM parameter, by analogy with a density clustering algorithm, and provide a method to estimate it. The FWHM is modified to account for the number of studies in the analysis, and represents a substantial change to the CBMA philosophy that can be applied to the current algorithms. Consequently we observe more reliable detection of clusters when there are few studies in the CBMA, and a decreasing false positive rate with larger study numbers. By contrast the standard definition (FWHM independent of the number of studies) is demonstrated to paradoxically increase the false positive rate as the number of studies increases, while reducing ability to detect true clusters for small numbers of studies. We also provide an algorithm for contrast meta-analysis, which includes a correction for multiple correlated tests that controls for the proportion of false clusters expected under the null hypothesis. Furthermore, we detail an omnibus test of difference between groups that is more sensitive than contrast meta-analysis when differences are diffuse. This test is useful where contrast meta-analysis is unrevealing.

## Introduction

A very popular method of performing a meta-analysis (MA) of functional magnetic resonance imaging (fMRI) and positron emission tomography (PET) data is coordinate based meta-analysis (CBMA). There are various approaches [1]–[10], but the common aim is to locate regions where different studies agree on the location of activation peaks (foci) better than expected by chance alone. Results are then thought to be of significance to the common functional aspect(s) of the studies included in the analysis. A further aim is to compare different groups, for example healthy control and patient groups, using contrast meta-analysis.

Here we focus on the activation likelihood estimate (ALE) based method, which is possibly the most widely known of the CBMA schemes. The ALE method models the uncertainty of the reported foci using a Gaussian function with specified full width half max (FWHM) [1]. It then estimates the likelihood, at each voxel, that there is consistent activation across multiple studies. Clusters of voxels with significantly high ALE are tested for by a permutation test. The ALE method is very popular, and has been, and is being, used to generate many publications. Despite this, there remain major problems.

The FWHM parameter, which is often set at ≈10 mm, has a major effect on the results [11]. In the similar kernel density analysis (KDA) method of CBMA, a FWHM of 10 mm or 15 mm is reported to produce the best results [7]. The signed differential mapping (SDM) uses 25 mm [5]. For the ALE methods, in an attempt to quantify the FWHM it has recently been estimated by fMRI experiment and a dependency on the number of subjects suggested [3]. Nevertheless, the lack of consensus on this parameter is one of the issues for CBMA. Indeed some CBMA methods remove the FWHM as a fixed parameter [9], [10]. However, these methods are sensitive to the required prior knowledge elicited from experts, and in the case of the method of Yue et. al. has not been generalised, in a computationally practical sense, to three dimensions.

Here a new FWHM scheme is introduced. This is motivated by the reasonable requirement that CBMA of a small set of studies should ideally produce results commensurate with those produced if the number of studies were increased. It redefines the FWHM as a density clustering parameter, rather than a specification of the uncertainty of the reported foci used by current ALE algorithms.

The correction for many correlated statistical tests is a problem for contrast meta-analysis that has not yet been addressed [12]; indeed contrast meta-analysis has previously been performed without any correction [13], which will inevitably lead to false positive results. Several methods are used to impose voxel-level (since testing is performed at each voxel) control of the rate of falsely rejected null hypotheses in CBMA; for example false discovery rate (FDR) control [4], [14]. The latest ALE algorithms have introduced cluster-level control to CBMA [2], which is preferred to voxel-level control since it directly relates to the results, by limiting cluster sizes to be larger than expected under the null hypothesis. CBMA is performed many 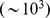 times using foci randomised throughout a brain mask and a user specified voxel-level threshold (for example 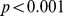 uncorrected). The distribution of the size of the resulting clusters is recorded, and a user specified quantile of this distribution (for example 95%) subsequently used as a lower permissible cluster size in the CBMA of the original foci. However, this scheme requires two independent user specified thresholds, so it is not clear exactly what this means for the proportion of falsely rejected null hypotheses. Furthermore, while this cluster-level control may be appropriate for CBMA where the null hypothesis is closely related to that obtained using random foci, it is not appropriate for contrast meta-analysis where the null hypothesis is obtained by permutation of the grouping variable.

We previously detailed a CBMA method that employed a false cluster discovery rate (FCDR) control scheme that has the particularly interpretable aim of limiting the proportion of the significant clusters expected under the null hypothesis. The control problem is further tackled by substantially reducing the number of statistical tests by testing only at the reported foci (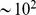 tests), rather than at each voxel (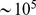 tests). We showed that this leads to fewer false positive results than using FDR correction at the voxel level, or using cluster-level control via minimum cluster size thresholding, yet maintains sensitivity [15]. Here we extend FCDR to contrast meta-analysis. The proposed method requires specification of only one threshold, is interpretable, and does not assume independence of the many tests.

The contrast meta-analysis method can localise significant focal differences between groups, indicating brain structures where functional activation differs. However, due to the need to correct for multiple comparisons, this method lacks sensitivity for detecting more diffuse subtle differences between the groups; such differences do not produce the very small p-values required to survive the correction. Lack of significant results from contrast meta-analysis is not, therefore, a good indicator that the two groups do not differ in activation pattern. Here we introduce an omnibus test of difference between groups. Only one test is performed, so no correction is necessary; this is similar to Fisher's combined probability test [16], but without the assumption for independence of the probabilities. Consequently it is better able to detect diffuse differences than contrast meta-analysis. The test can provide evidence for difference between groups when the contrast meta-analysis is unrevealing.

In summary, this report describes new tools for ALE based experiments. Specifically: 1) a new definition and method for setting the FWHM parameter, 2) a description of the contrast meta-analysis algorithm and subsequent correction for multiple correlated comparisons at the cluster-level, and 3) an omnibus test of difference between groups.

## Materials and Methods

Statistical significance in the ALE method is judged relative to a null distribution of ALEs, generated by permutation of the foci throughout a grey matter (GM) mask [2], [7]. When many studies report activations in similar locations, the foci density is high so there is little distance between them. In regions reported as active by few studies, the foci are lower in density.

While our algorithm (LocalALE) has been detailed previously [15], some specifics are important to the methods presented in this report. The ALE method models the spatial distribution (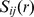) of the 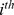 reported foci in the 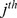 study (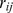) by a Gaussian distribution of specified standard deviation (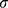) or FWHM. The Gaussians are truncated at 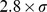, or equivalently 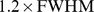,



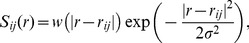
(1)where



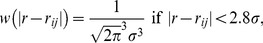
(2)and is zero otherwise. The truncation removes 5% of the ‘mass’ of the Gaussian, and is performed to reduce the influence of foci over long distance, and to make testing only at the foci possible [15]. It has previously been shown that with 5% truncation, the resulting clusters are similar to those obtained using the ‘full’ Gaussian [15]; clearly some truncation is always present even with the ‘full’ Gaussian, imposed by the brain volume. The spatial activation distribution for study 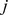 is



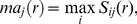
(3)from which the ALE is computed.

Foci separated by a distance 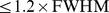 overlap (are separated by less than the truncation distance). Overlapping foci across studies form clusters, which, if significant, are the results of the CBMA.

The software is available to download and use freely from www.nottingham.ac.uk/research/groups/clinicalneurology/neuroi.aspx.

### The FWHM in ALE based CBMA

The FWHM can affect the results of ALE based CBMA [11]. Often the FWHM is set to 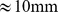, but there is some variance on this. An empirical FWHM has been described [3], based on the idea that it measures the variance of activation peak position on repeating the same experiment in a group of volunteers. This suggests that the FWHM should depend on the number of subjects within a study; the larger the number, the smaller the uncertainty in the location of the peak of the activation, and so the smaller the FWHM. While this is intuitively reasonable, it does create some issues. Firstly, the suggested estimate is based on a specific functional experiment, and might not be generalizable. Secondly, ideally the results from a small CBMA study should agree with a larger CBMA performed if more studies were available. In this report a new definition and estimate for FWHM that depends on the number of studies in the CBMA is presented.

**Figure 1:**
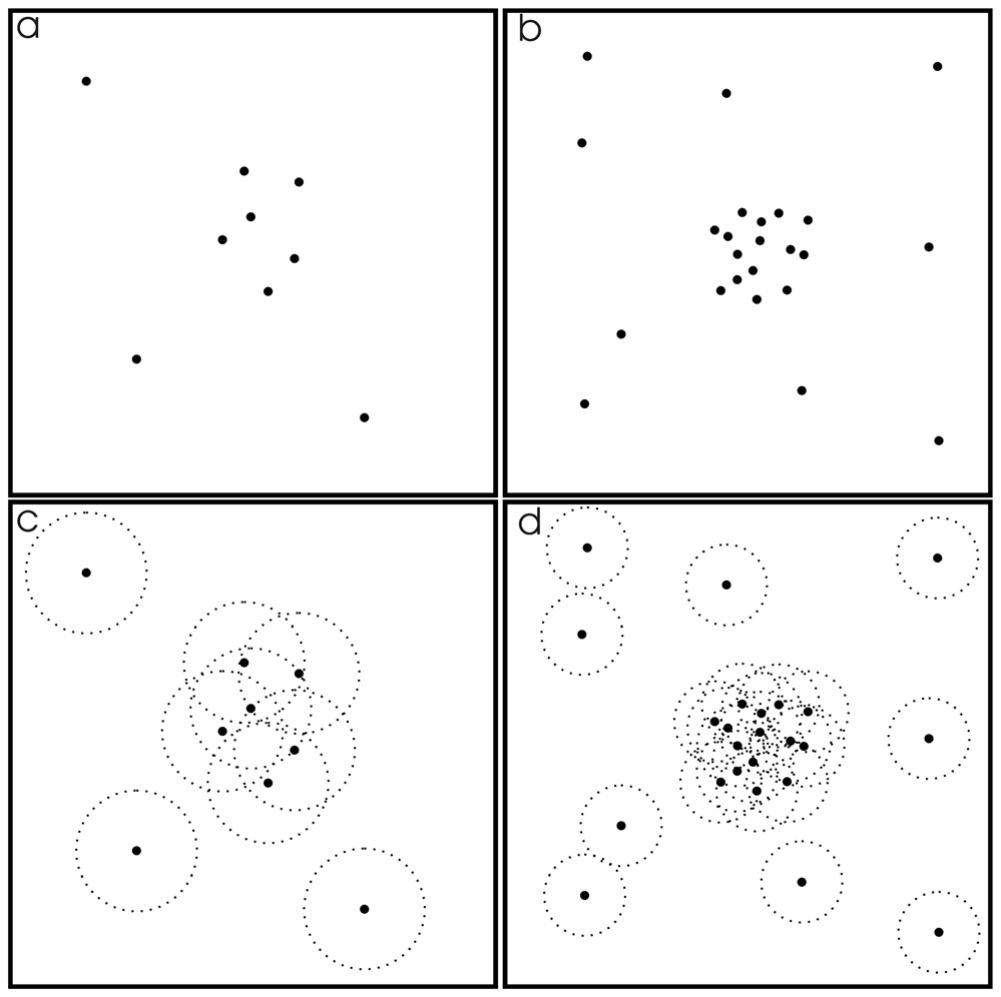
Depicts how the Eps parameter in the density clustering algorithm DBSCAN is modified as the size of the data set is changed. For sparse data sets (a) the Eps parameter is large (represented by the radius of the circles in (c)). For the larger data set, Eps is smaller. Eps is chosen so that the dense cluster of points and the noise are separate. This clustering algorithm is analogous to CBMA, and the Eps parameter analogous to the FWHM.

Each study in the CBMA reports foci of activation, some of which are common to a proportion of the studies, while others are due to some study specific aspect. Where there is better than chance agreement across studies about the activation peaks, the foci form clusters of higher than average density. Between the clusters, the study specific foci form a lower than average density noise. Identifying clusters of high density points in the presence of noise can be tackled using the density-based spatial clustering in applications with noise (DBSCAN) [17] algorithm. Clusters in DBSCAN are formed by points that are density reachable, such that any point in the cluster can be connected to any other within the cluster via a chain of points that are separated by a distance less than parameter ‘Eps’. The problem is depicted in figures 1a and 1b; figure 1a shows a sparse set of points and figure 1b a larger set of points. If Eps is set too large, the clusters begin to merge, and the noise points are recruited to the clusters. If Eps is set too small, the clusters are missed. In figures 1c and 1d Eps is represented by the radius of the dotted circles. For the algorithm to succeed the Eps parameter must be set such that within-cluster the points fall within a distance Eps of other points in the cluster, while the noise must be beyond this distance. Consequently, for the sparse set of points (figures 1a and 1c), Eps is larger than for the larger set of points (figures 1b and 1d).

Since the density clustering problem is analogous to CBMA, it is proposed that the FWHM parameter be redefined to be analogous to the Eps parameter in density clustering. The aim is to increase the FWHM for small numbers of studies such that the within-cluster foci can overlap, and reduce the FWHM for larger numbers of studies to prevent the study specific foci joining the clusters. To achieve this it is proposed that the characteristic volume occupied by each foci 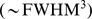 is inversely proportional to the number of studies such that



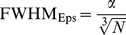
(4)where *N* is the total number of studies, and 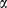 is a constant to be estimated. The proposed estimate of FWHM is now 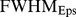 to signify its relationship to both the FWHM conventionally used in CBMA and the clustering parameter used by DBSCAN. The parameter 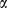 can be estimated under the null hypothesis (randomised foci) where the average density of the foci across studies is higher than the study specific foci, and lower than the clustered foci. It is hypothesised that this scheme will: 1) help to prevent incidental recruitment of study specific foci to the clusters, and 2) help with the detection of low density clusters when there is little data.

This constitutes a redefinition of the FWHM parameter. It no longer represents spatial uncertainty in the location of the foci, but rather it is a density parameter similar to Eps, used in the DBSCAN [17] algorithm.

#### FWHM Experiments

The effect of varying the FWHM between 6 mm and 18 mm is visually assessed, using LocalALE, in three sets of fMRI data: 1) the pain data (47 experiments) used in [15], 2) n-back data (61 experiments) used in [18] and downloaded using Sleuth (downloadable from www.BrainMap.org) from the BrainMap database [19], and 3) Stroop test data used in [20] and again downloaded using Sleuth; workspace files for downloading the data via Sleuth are available from the BrainMap website. It is expected that if the FWHM is too small, the clusters will be small, or even vanish. It is also expected that if the FWHM is too large, the clusters will merge and expand in spatial extent by including more nearby foci. In the case of the Stroop experiment, the data are separated into two groups: pooled studies requiring either a verbal response or a mechanical response (19 studies), and those specifically requiring mechanical response (6 studies). From the original meta-analysis it is expected that there is some similarity between these, and certainly this is expected on the grounds that one is a subset of the other. The difference in numbers of experiments in the two groups is used to demonstrate how FWHM must be modified to make the results commensurate.

The mean effect of FWHM on the clustering under the null hypothesis is also investigated; under the null hypothesis it is expected that large clusters do not form on average. Foci are randomised throughout a grey matter mask, as detailed in [15], 10 times. For each randomisation the number of clusters (sets of foci in different studies separated by a distance smaller than 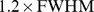) is counted. The mean number of clusters, as a proportion of the total number of foci, is plotted against FWHM to see when the clusters begin to form. It is expected that for very small FWHM the foci in different studies do not overlap under the null hypothesis, so the number of clusters will be equal to the number of foci; each non-overlapping focus being considered a cluster of one focus. As the FWHM is increased, foci will start to overlap and form larger clusters. An empirical estimate for parameter 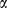 (equation (4)) is obtained by identifying the point where clusters just begin to form under the null hypothesis.

Two large data sets used previously [21] to examine the functional connectivity of two structures, the right amygdala (RA; 189 studies) and the orbitofrontal cortex (OFC; 142 studies), are explored. The data sets are available for download from www.BrainMap.org, and were originally created by searching the BrainMap database [22] for any studies that report at least one activation within a seed region of interest (ROI); the ROIs are depicted in [21]. These datasets are of interest since it is obvious that there are significant clusters where the seed ROIs are defined, and that those clusters should be similar in size to the seed ROIs because they determine where the foci are located. Coordinate based meta-analysis is performed on the full datasets, and also on smaller subsets of 25 (25 is arbitrary, but not an unusual size for a typical CBMA experiment) randomly selected studies from each full dataset. The proposed 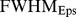, and the FWHM specified in [3] and incorporated into GingerALE (a popular and freely available program used for ALE analysis; available from www.brainmap.org) are compared; GingerALE is used to demonstrate that the sensitivity to FWHM is not specific to our LocalALE method. It is expected that the clusters in the RA and OFC should be similar in the small and full datasets.

Finally a numerically generated pseudo experiment is created. Each pseudo study in the experiment has 10 foci (around 10 foci would not be unusual in a real study), which are placed either within one of three clusters, or at random with uniform probability within the GM mask but outside of the clusters. The experiment is performed twice: a) with the proportion of studies reporting in the three clusters being 40%, 50%, and 60%, to reflect the proportion ranges observed in most significant clusters from the pain, Stroop, and n-back experiments, and b) with the proportion of studies reporting in the three clusters being 20%, 30%, and 40%, to consider the impact of less consistent activations. Foci that are truly members of the 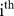 cluster are placed at location 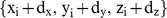, where d are randomly generated from a truncated (at 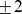 standard deviations from the mean) Gaussian distribution with mean zero and FWHM = 10 mm. The results of CBMA on these experiments are depicted for: 1) 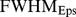 using 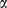 (equation (4)) estimated from the pain, n-back, and Stroop experiments, 2) FWHM = 10 mm, and 3) using the FWHM estimate detailed by [3] and employed in GingerALE.

True and false positive rates are explored quantitatively for this numerical experiment as a function of the number of studies (10 to 150 studies); 100 averages are performed for each number of studies. The true positive rates are the proportion of true cluster foci that are declared as cluster members, while the false positive rates are the non-cluster foci declared as members of a cluster. Both true and false rates are expressed as a proportion of the total number of true cluster foci to make them easy to compare. If the clustering analogy is valid for our CBMA experiment, it is expected that with FWHM = 10 mm there will be fewer true positive results for few studies compared to 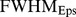. Furthermore, it is expected that for large numbers of studies that the false positive rate will be higher for FWHM = 10 mm than for 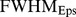.

### Contrast meta-analysis

Contrast meta-analysis attempts to find differences in activation pattern between two groups of studies. The results are clusters of foci where there is localised significant difference in ALE between the groups. The null hypothesis is that there is no difference between the groups, so a permutation (of the group variable) test is employed. Following on from our LocalALE algorithm, tests for differences are performed only at the foci in the method reported here; instead of at each voxel. It also employs the false cluster discovery rate (FCDR) control of false positive results, as detailed previously in [15]. FCDR is particularly interpretable since it controls for the proportion of significant clusters expected under the null hypothesis. It also takes account of the correlated nature of the tests; the ALE values, and p-values, for each focus depends on the other foci and are therefore not independent. Contrast meta-analysis is useful when there are very significant localised differences between the groups that can survive the FCDR control.

Studies are separated into two groups, *A* & *B*, containing 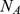 and 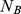 studies, respectively. Of all the permutations, the 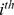 is specified by



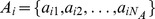
(5)and



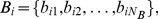
(6)where 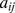 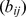 are the study numbers, and 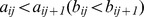 since order is not important. The ALE for group *A*, permutation *i*, at location *r* is



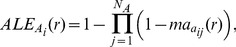
(7)and similar for group *B*. The difference in ALE between the two groups is



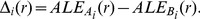
(8)


For a particular permutation, *k*, and for a particular focus 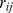, the significance (p-value) of the difference in ALE between the groups is



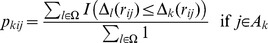
(9)or



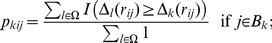
(10)

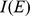
 is an indicator function that equals 1 if *E* is true, and zero otherwise. A small value of 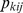 indicates that the magnitude of the ALE difference for foci 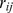 is particularly large for the 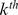 permutation. The sums in these expressions are over the set (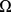) of all possible permutations of the grouping. However, since 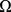 is typically very large, p-values are estimated using a random selection of 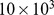 permutations. Here, as suggested previously [13], statistical tests are performed only for foci that are found to be significant by CBMA. This reduces the number of tests, and so increases the power, at the expense of testing for differences between the complete groups.

It is hypothesised that the groups 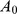 and 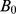, which might be patients and healthy controls for example, have localised differences in activation pattern. The p-values 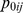 measure the significance of that difference. Clusters of significant foci are found, and counted, using the clustering algorithm provided in the supplement (File S1); the algorithm finds the most significant foci first then uses Dijkstra's algorithm [23] to locate all other foci in the cluster, repeating the process to locate all clusters. The number of significant clusters is 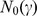, which is computed using only the foci with 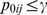. To control the FCDR, an estimate of the number of clusters expected under the null hypothesis is needed. This is estimated using a randomly selected set of 2000 permutations 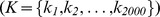, computing the number of clusters for each of these permutations 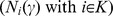 and averaging; 2000 is considered sufficient as repeating the experiments gives similar estimates of FCDR. Controlling the FCDR at a level of 0.05 (for example) is then performed by maximising 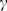 such that



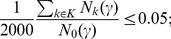
(11)the proportion of clusters expected under the null hypothesis is, at most, 0.05 with this 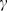. Further detail about the calculation of FCDR is given in [15].

#### An omnibus test for difference between groups

Contrast meta-analysis looks for differences in activation patterns, between groups, by location. But such analysis requires very significant differences; since the p-values need to be very small to remain significant after controlling for many statistical tests. An omnibus test can provide evidence of pattern differences when contrast meta-analysis produces no significant results. Such a test may be useful for detecting differences that are subtle, but spread across substantial regions of the activation pattern.

A log likelihood value can be computed using the p-values from equations (9) and (10),



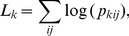
(12)where the sum is over all foci in permutation *k*. The magnitude of this will be small under the null hypothesis and larger when the data are critical of the null hypothesis. The distribution of 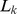 is not known because the p-values are not independent (for independent p-values, Fisher's combined probability test can be used), but can be estimated using a random selection of permutations 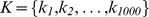. Only 1000 permutations are used, and found to be sufficient, to estimate the single p-value for the omnibus test



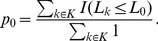
(13)


This test is performed including all foci in the experiment.

#### Experiments for contrast analysis

To explore the two different scenarios considered above (highly significant local differences between groups, and more subtle wide spread differences) numerical experiments have been devised.

The first experiment involves two groups of studies with a cluster reported at {34, 10, 16}mm (Talairach coordinates) in group *A* and {−34, 10, 16}mm in group *B*. A further eight clusters are reported similarly by both groups. All foci that form part of a cluster have a random spatial perturbation as described for the FWHM numerical experiment. From the pain and n-back experiments, about 50% of studies report at the site of the most significant clusters. Therefore, each of the clusters in this experiment contains foci from half of the studies, and half of the foci in each study are distributed randomly, and with uniform probability, throughout the GM mask.

The two clusters at 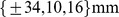 should be highly significant with sufficient studies in the experiment. The clusters that are reported similarly by both groups should not produce significant differences. Results are reported in a table indicating number of clusters reported by both groups, number of studies per group, and significances by contrast meta-analysis and the omnibus test.

The second experiment involves up-to sixteen clusters reported by both groups. In group *A* the clusters involve foci (with the random perturbation used in the first experiment) from 50% of the studies, and half of the foci in each study will be randomly distributed, with uniform probability, throughout the GM mask. Group *B* is similar, but the proportion of studies reporting at each cluster is lower. The differences between the studies are then subtle, and spread across the activation pattern. Results are reported in a table indicating number of studies, proportion of studies reporting at clusters, and significances by contrast meta-analysis and the omnibus test.

The contrast meta-analysis and omnibus tests are performed using 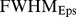.

## Results

### FWHM experiments

#### Assessment of the effect of the FWHM parameter on real data

**Figure 2:**
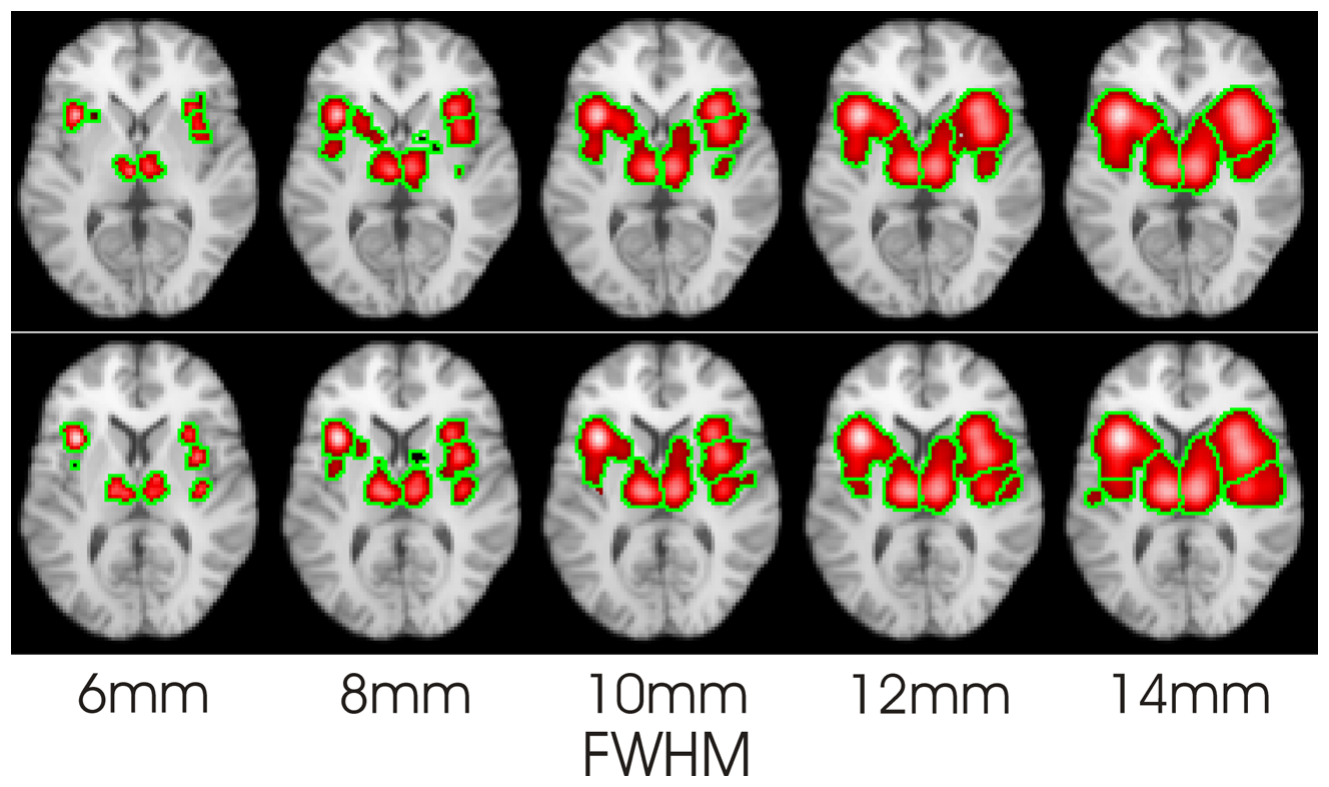
CBMA results for the 47 studies included in the pain CBMA for a range of FWHM produced using LocalALE. With small FWHM, the clusters shrink, or vanish. With large FWHM, the clusters merge and expand.

Figure 2 shows the result of CBMA of 47 pain studies performed on healthy subjects. Result are shown for two different slices (top and bottom rows) using FWHM ranging from 6 mm to 14 mm. For very small FWHM the clusters (outlined in green) become fragmented and small. At larger FWHM the clusters begin to merge and grow.

**Figure 3:**
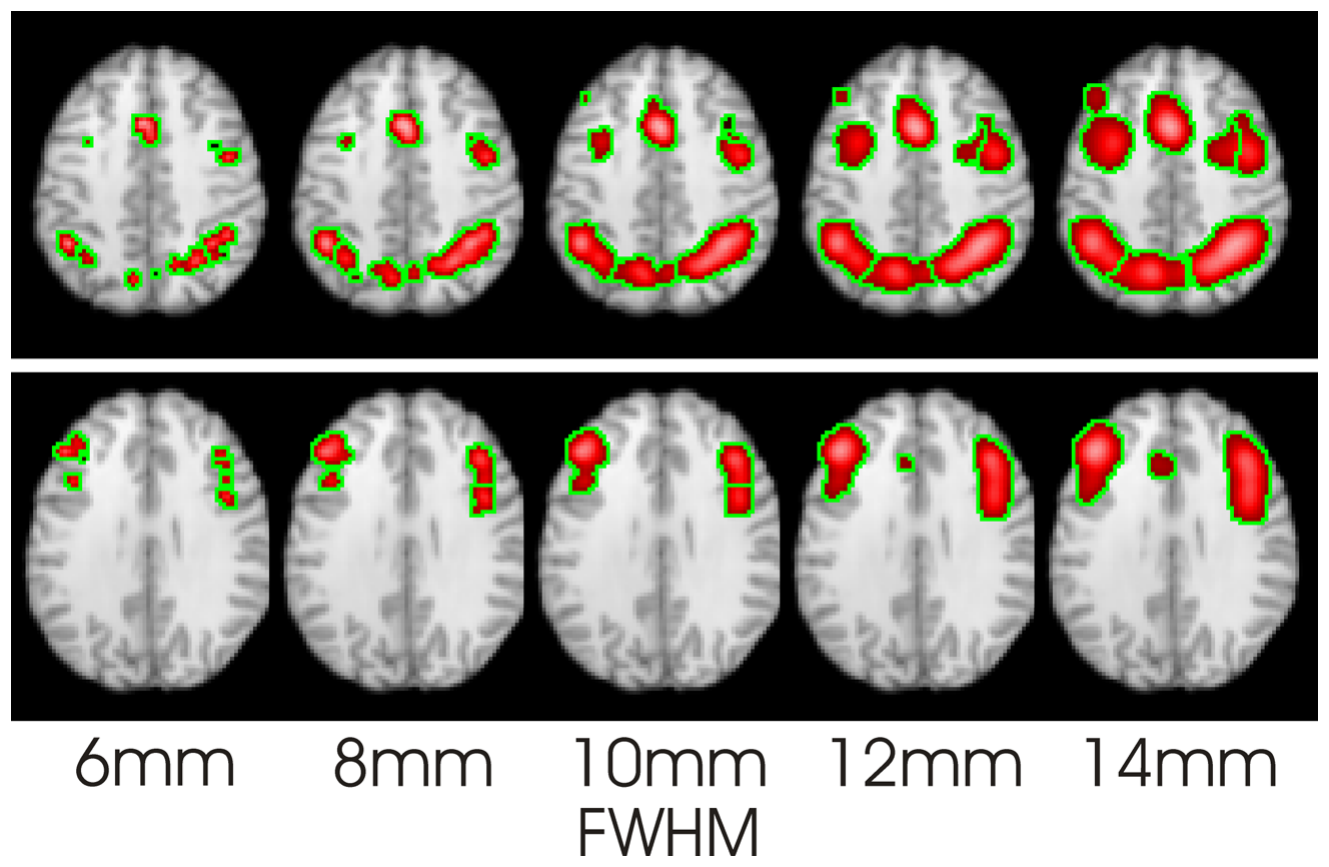
CBMA results for the 61 studies included in the n-back CBMA for a range of FWHM produced using LocalALE. With small FWHM, the clusters shrink, or vanish. With large FWHM, the clusters merge and expand.

Figure 3 shows the CBMA results for the 61 n-back studies. Two different slices are shown (top and bottom rows) using FWHM ranging from 6 mm to 14 mm. As expected, for very small FWHM the clusters become fragmented and small. At larger FWHM the clusters begin to merge and grow.

**Figure 4:**
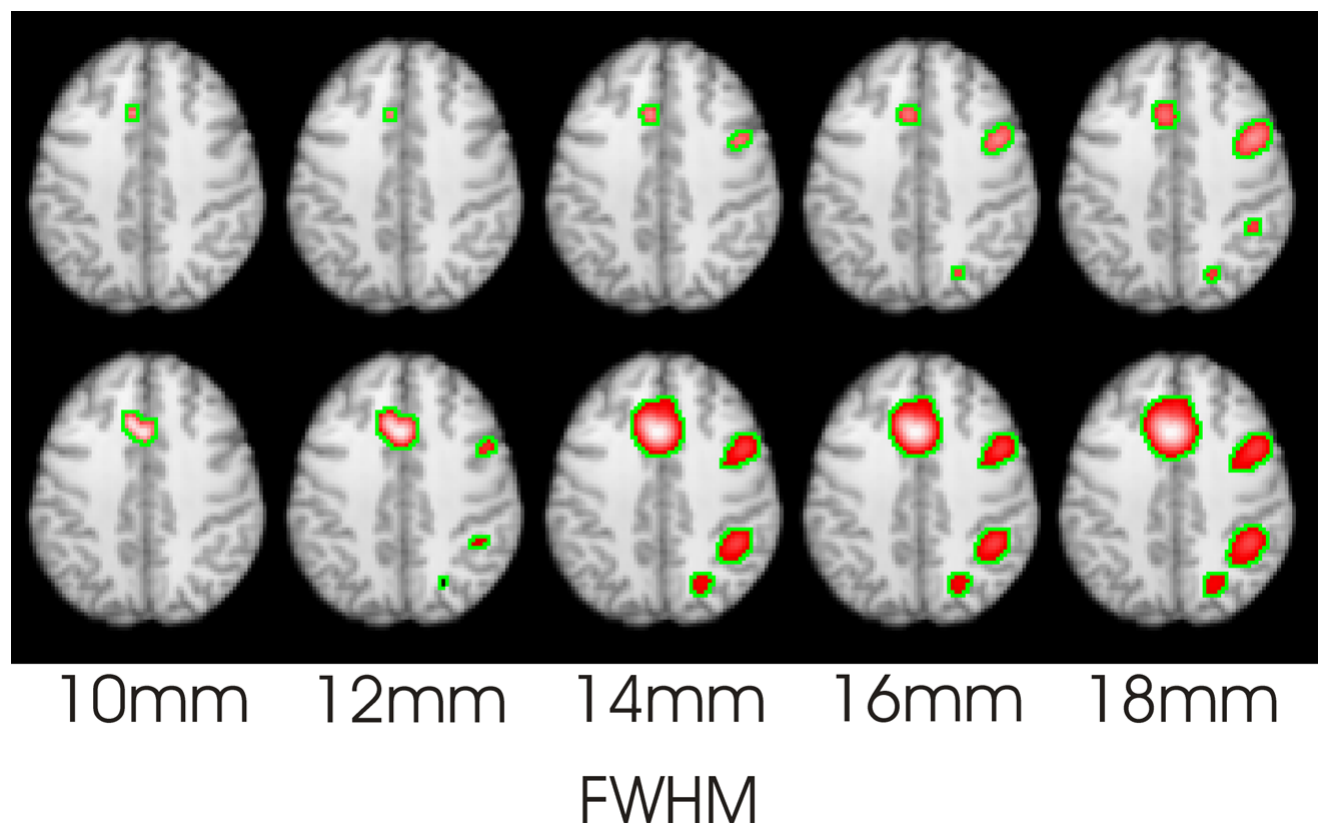
CBMA results for the Stroop experiment, for a range of FWHM produced using LocalALE. Top row shows the Stroop studies where manual response was required (6 studies). Bottom row shows the pooled Stroop studies (19 studies). The studies give similar activation patterns, but only if the FWHM is modified to account for the number of studies per experiment.

Figure 4 shows the CBMA results for the 6 manual (top row) response and 19 pooled (bottom row) Stroop studies. The results are similar, but at different FWHM. Below 18 mm in the manual Stroop experiment, the clusters vanish or shrink. Above 12 mm in the pooled stroop experiment the clusters just increase in size.

#### Estimating the parameter 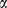


**Figure 5:**
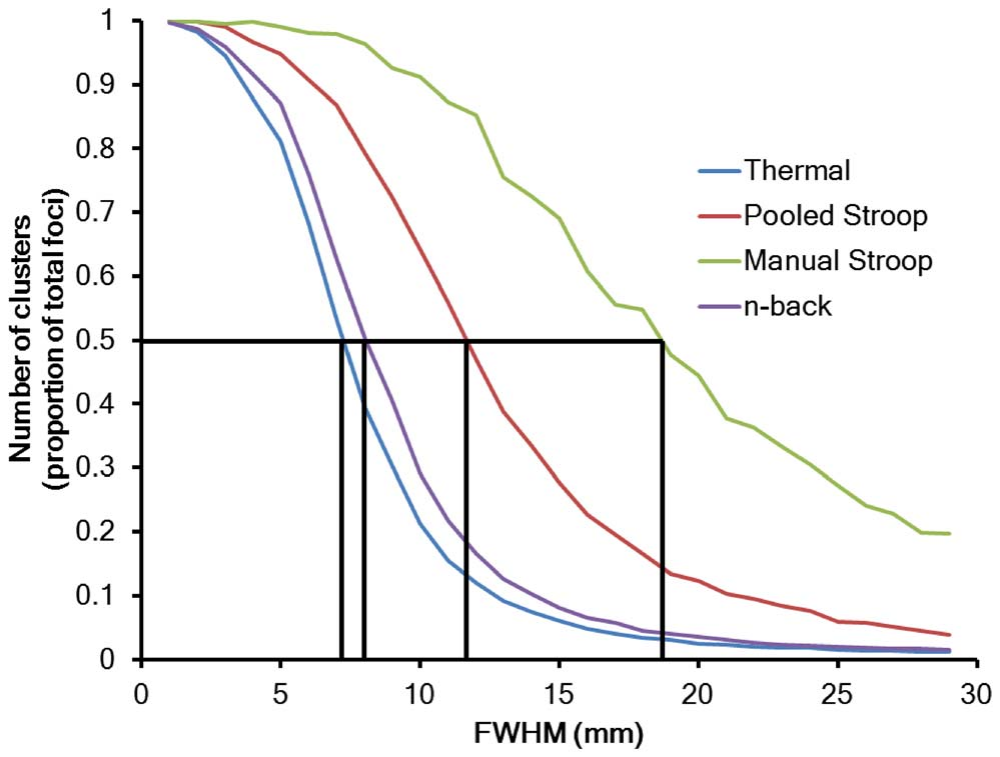
Depiction of cluster forming under the null hypothesis (randomised foci) as a function of FWHM. For small FWHM, the foci do not overlap, so there are as many clusters as foci (single focus clusters). As the FWHM increases, larger clusters begin to form.

Figure 5 depicts the number of clusters, under the null hypothesis (foci randomised), for each experiment as a function of FWHM. Also indicated are the FWHM where clustering just begins (number of clusters = 0.5) under the null hypothesis for the pain, n-back, and Stroop experiments. Higher FWHM generates larger clusters, which is not expected under the null hypothesis.

An estimate of 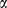 can be obtained from figure 5. If the 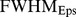 is the parameter value that just starts to form clusters under the null hypothesis, such that the foci overlap on average with one other, then the intercept of the curves with 0.5 on the *y* axis gives FWHM estimates of around 7 mm, 8 mm, 12 mm, and 18 mm for these experiments. Given the number of studies in the experiments, and using equation (4), yields an estimate for 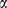 of about 30 mm. The 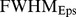 estimates are then 8.3 mm, 7.6 mm, 11.2 mm, and 16.5 mm for the pain, n-back, pooled Stroop, and manual Stroop studies respectively.

**Figure 6:**
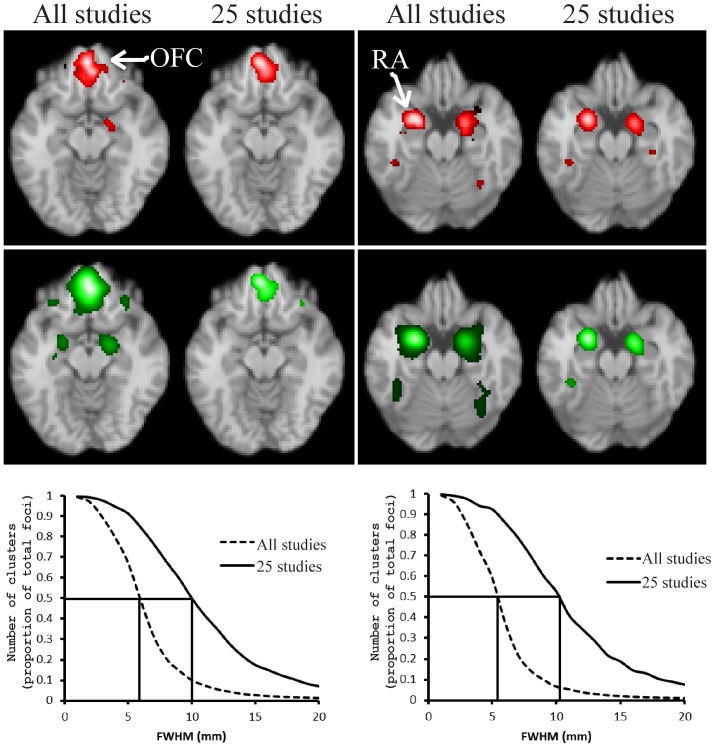
CBMA of the orbitofrontal cortex (OFC) and right amygdala (RA) studies, and smaller sub-studies. The top row depicts the results using 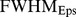, the middle row is the result from GingerALE, and the bottom row depicts the number of clusters, as a function of FWHM, counted under the null hypothesis.

Figure 6 shows the results from CBMA of the RA and OFC experiments. Using 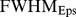 (top row), with 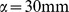, produces clusters that are quite independent of the number of studies. Furthermore, the clusters are of a reasonable size given the seed ROIs presented in [21]. When the FWHM is determined as detailed in [3] (middle row), the clusters are quite large for the complete data sets, particularly considering the seed ROIs. The cluster sizes are also quite dependent on the number of studies. The number of clusters expected under the null hypothesis, as a function of FWHM, is shown on the bottom row. Estimates of a obtained at the point where the foci just begin to cluster (intercepting the *y* axis at 0.5) are 29 mm, 29 mm, 29 mm, and 31 mm for these four experiments.

#### Numerical simulation experiments

**Figure 7:**
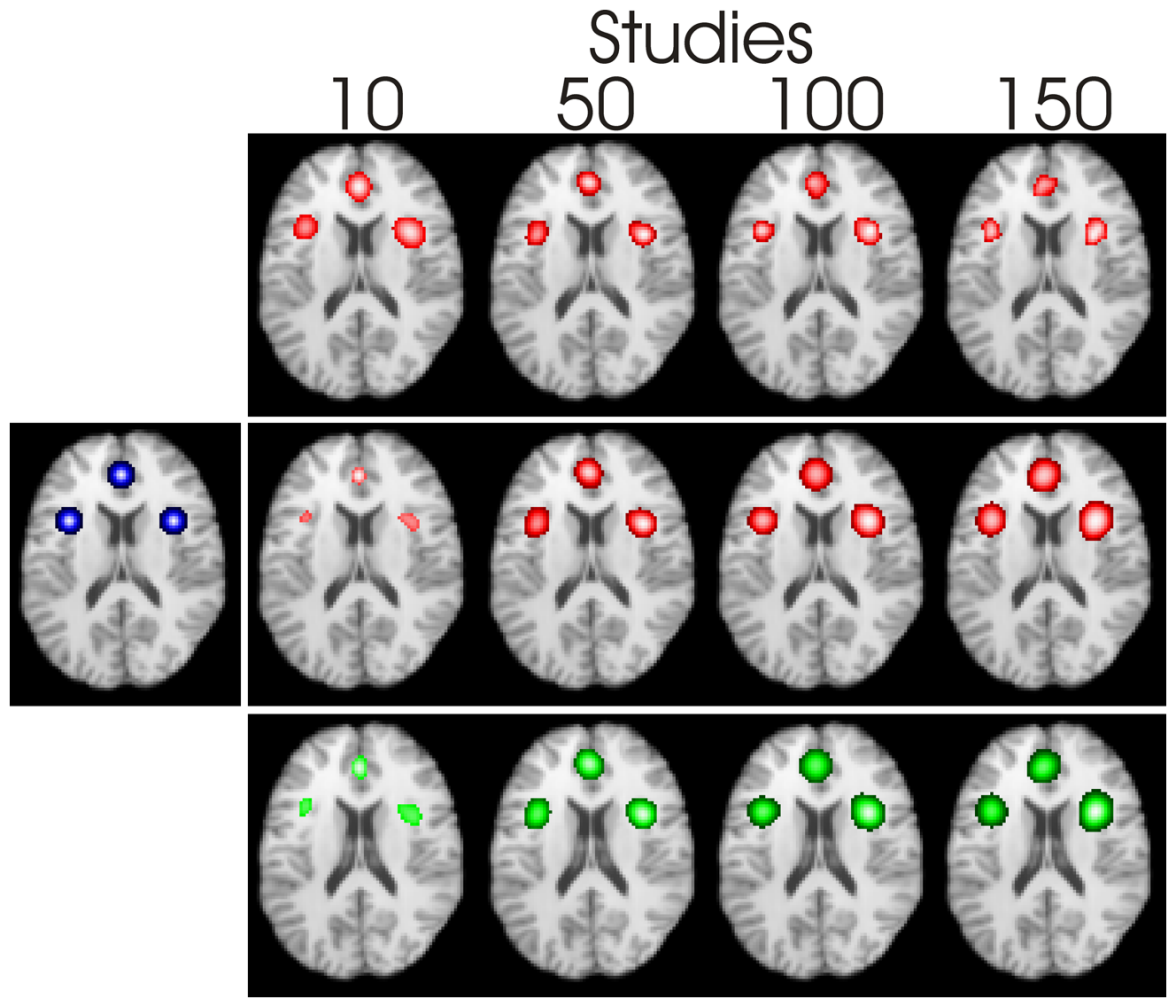
CBMA results for various numbers of studies, and using three definitions of the FWHM: top row uses 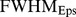, middle uses FWHM = 10 mm, and bottom uses the method reported by [3]; produced using GingerALE. The left-most image shows the distribution function of the foci as a blue overlay.

Figure 7 shows the CBMA results for the simulated experiments; only the experiment with 40%, 50% and 60% of studies reporting in the clusters is shown as the lower proportion experiment is visually similar. The number of studies per experiment clearly has an impact on the results when using fixed FWHM; middle (FWHM = 10 mm) and bottom (FWHM as defined in [3] and produced using GingerALE) rows. When using 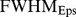 with 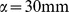 in equation (4) (top row) the results are not so dependent on the number of studies. Furthermore, the cluster size, using 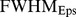, is the most consistent with the foci distribution function, shown as a blue overlay on the left-most image; with the other FWHM estimates the clusters are small with just 10 studies, but expand in size beyond the boundary of the distribution function for large number of studies.

**Figure 8:**
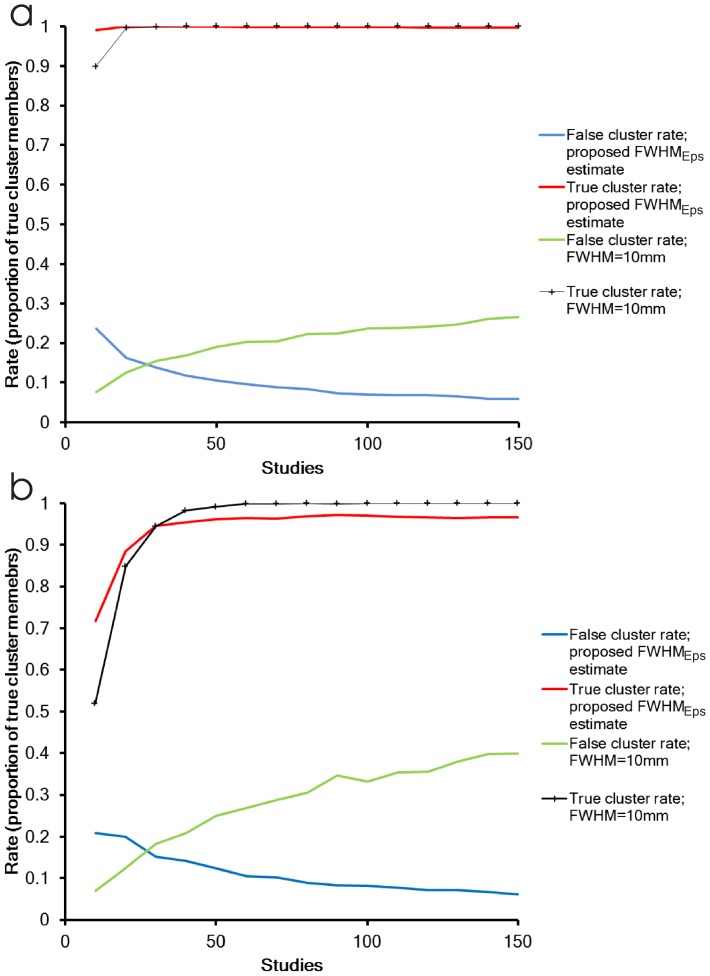
True and false cluster rates for 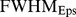 and for FWHM = 10 mm. a) shows rates when the proportion of studies reporting in the clusters is 40%, 50%, and 60%. b) shows the rates when the proportion of studies reporting in the clusters is 20%, 30%, and 40%.

Figure 8a explores the true and false positive rates associated with the numerical experiment where the proportion of studies reporting at the clusters is in the range 40% to 60%; both rates expressed as a proportion of the true cluster members. True cluster rates were very high regardless of the FWHM estimate used, except for ten studies where the larger 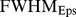 estimate is able to detect a greater proportion of true cluster members. This, however, is associated with an increase in the false cluster rate, since the larger 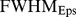
will also capture some of the study specific foci, which is noticeable in figure 7. More striking are the false cluster rate trends in this plot. For a FWHM of 10 mm, the false cluster rate is increasing with the number of experiments. This leads to the paradoxical observation that increasing the number of studies included in the meta-analysis can be detrimental to the results. Using 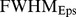 results in a reducing false cluster rate with increasing study numbers, which is sensible for a meta-analysis; a fit revealed that the false cluster rate was 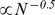 for this experiment.

Figure 8b explores the true and false positive rates associated with the numerical experiment where the proportion of studies reporting at the clusters is in the range 20% to 40%. In this case the true cluster rate is reduced when there are few studies because the FWHM is not large enough to cause clustering. The impact of this is reduced in the proposed method, which has a larger FWHM for fewer than 30 studies, but at the expense of higher false cluster rates. For larger numbers of studies the true cluster rate reaches 100% for FWHM = 10 mm, but is slightly lower (97%) for the proposed method due to its smaller FWHM. Once more the most striking trends are those of the false cluster rates. While using 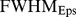 reduces this as the number of studies increases (again the rate was 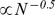), it is increased when the FWHM is fixed at 10 mm.

These results are not specific to LocalALE, as we have shown in figure 7. Indeed the contribution list for each cluster provided by GingerALE confirmed that a very high proportion of studies were reported falsely. Adjustment of the FWHM to account for the number of studies is therefore suggested.

#### Contrast analysis experiments

**Figure 9:**
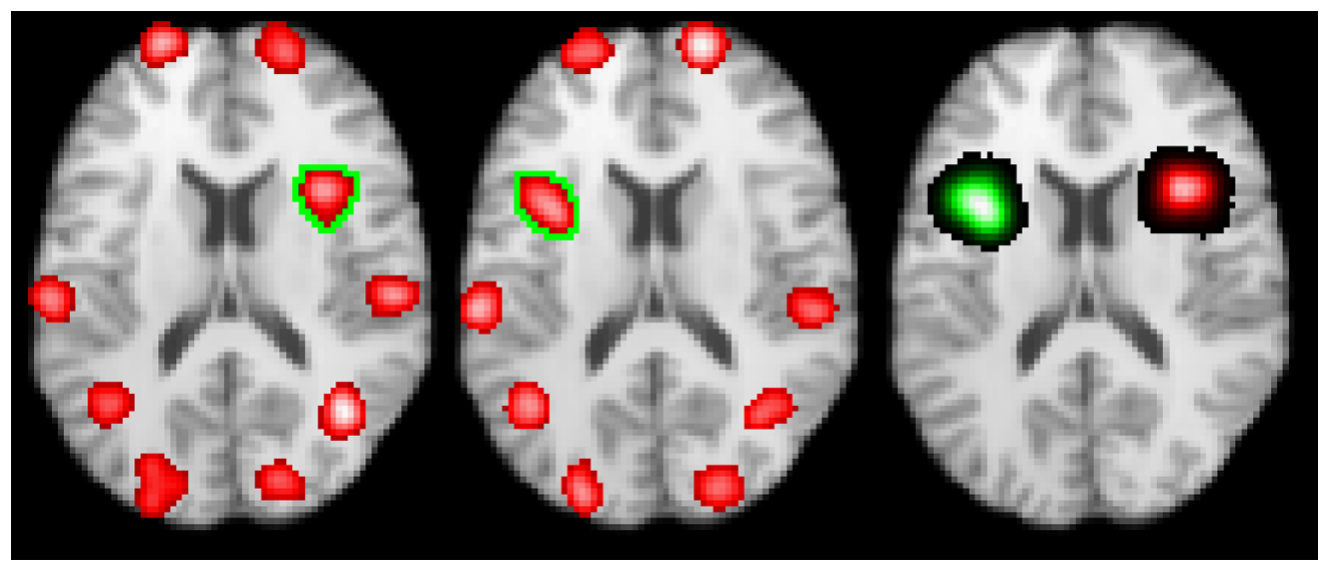
CBMA results of the first contrast meta-analysis experiment. The left and middle images show groups *A* & *B*, which have eight clusters in common, and two (one each) group specific clusters. The right image shows the difference between the groups found using contrast meta-analysis. This experiment tests the ability of contrast meta-analysis and the omnibus test to detect very significant differences between groups in the presence of an otherwise similar activation pattern. Note that while an intensity threshold (the lowest significant ALE value) is applied the leftmost and middle images, no such threshold is applied to the ALE difference image (right).

Figure 9 shows the clusters used for the first contrast meta-analysis experiment. The eight clusters reported by both groups are seen in the left and middle image, along with the group specific clusters; highlighted by green ROIs. The rightmost image shows the contrast image, indicating where the activation patterns differ. In both groups 50% of studies report in each cluster.

Table 1 shows that the results of contrast meta-analysis are not significant (FCDR>0.05) for 10 or 14 studies per group, and just about significant with 16 studies per group. When contrast meta-analysis was significant, it managed to find both group specific clusters, as expected. The omnibus test is significant in all of the experiments, indicating greater sensitivity. This is because the omnibus test needs no correction for the multiple tests. It should be noted that the p-values and FCDR values reported in table 1 are approximate; partly because they are estimated by permutation test, but mostly because the experiments include random foci, which would be different if the experiment were re-generated. Experiments were therefore repeated several times to check that the results presented were representative.

**Table 1:** Results for the first group comparison experiment.

Number of studies per group	Results of contrast meta-analysis	Omnibus test using all foci (p-value)
10	0.4	0.04
14	0.1	0.02
16	0.05	0.01
20	0.02	0.002

The omnibus test is able to find differences between the two activation patterns (see figure 9), even when the contrast meta-analysis test is unrevealing. The two tests are more sensitive for larger numbers of studies.

**Figure 10:**
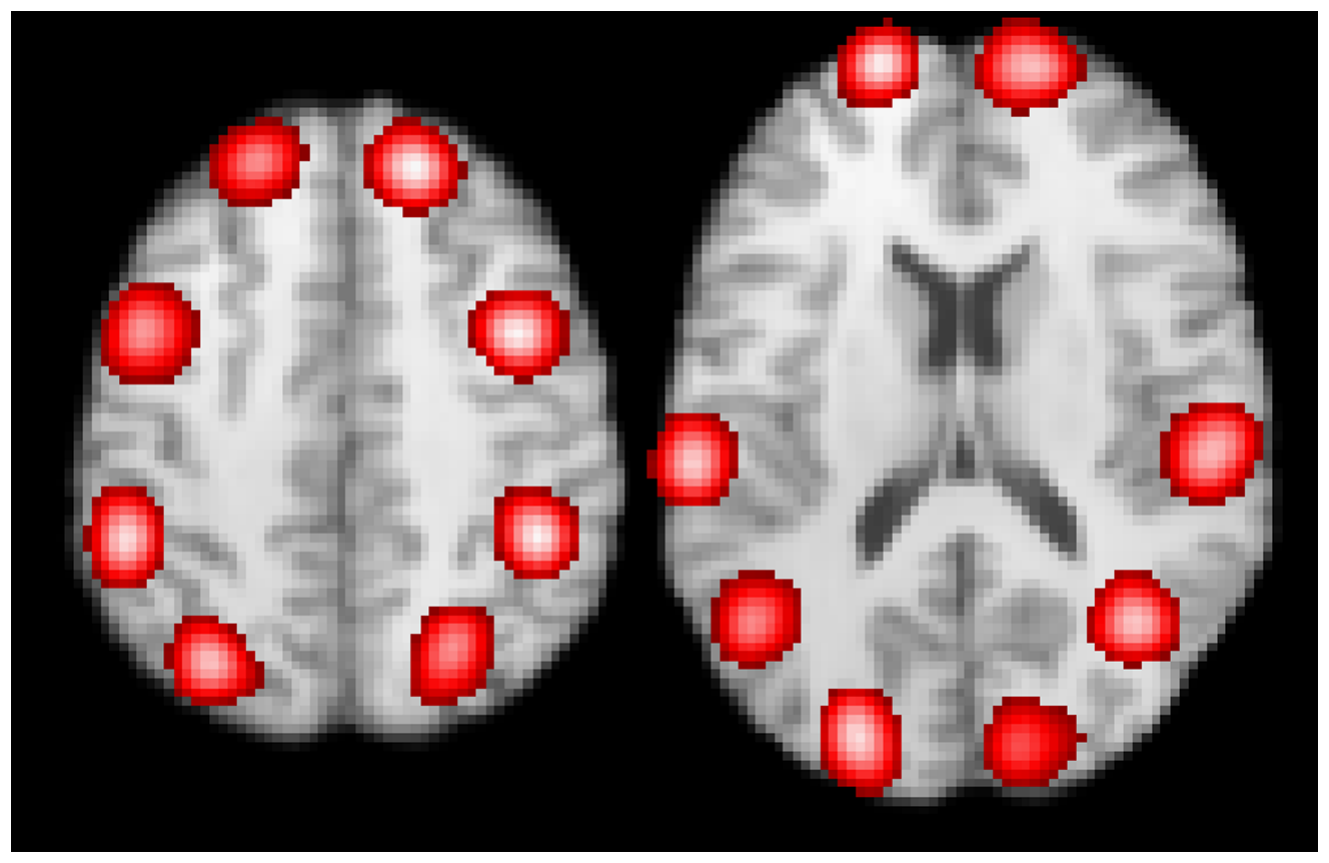
CBMA results of the second contrast meta-analysis experiment. Each group reports the same number of clusters (up-to 16, as shown), but with different frequencies. This experiment tests the ability of contrast meta-analysis and the omnibus test to detect subtle differences spread across the activation pattern.

Figure 10 shows the 16 clusters used for the second contrast meta-analysis experiment. Both group *A* and group *B* report some, or all, of these clusters. In group *A* 50% of studies report in each cluster, while a smaller percentage of studies in group *B* report at each cluster (see table 2).

**Table 2:** Results for the second group comparison experiment (see figure 10).

Number of studies per group	Number of clusters in experiment	Number of foci per group	Number (%) of studies reporting at clusters in group A	Number (%) of studies reporting at clusters in group B	Number of clusters found by contrast meta-analysis with FCDR≤0.05	Omnibus test using all foci (p-value)
6	8	48	3 (50%)	1 (17%)	0	0.03
6	16	96	3 (50%)	1 (17%)	0	0.03
10	8	80	5 (50%)	2 (20%)	0	0.01
10	8	80	5 (50%)	1 (10%)	0–6	0.004
10	16	160	5 (50%)	2 (20%)	0–1	0.02
20	8	160	10 (50%)	4 (20%)	0	0.001
20	8	160	10 (50%)	2 (10%)	5–8	0.001
20	16	320	10 (50%)	4 (20%)	0–4	0.001
40	8	320	20 (50%)	8 (20%)	0	0.001
40	8	320	20 (50%)	4 (10%)	7–8	0.001
40	16	640	20 (50%)	8 (20%)	4–16	0.001

The omnibus test is able to find differences between the two activation patterns, even when the contrast meta-analysis test is unrevealing. The two tests are more sensitive for larger numbers of studies, larger differences per cluster, or more clusters that are different.

Table 2 shows the results of contrast meta-analysis and the omnibus test when differences between groups are spread over the activation pattern. As the differences between the studies increase, either per cluster or as the number of clusters that are different increases, the tests become more sensitive as expected. However, contrast meta-analysis is not completely successful in finding all differences between the two groups, as indicated by the range of clusters found on repeating the experiment; with different random foci. The omnibus test is certainly more sensitive, able to detect differences between even small groups of studies. It should be noted that the smallest p-value reported for the omnibus test is 0.001; since only 1000 permutations are used to compute the p-value, 0.001 is the smallest non-zero value possible.

## Discussion

We have detailed three tools for use with coordinate based meta-analysis. By analogy with density clustering, we have redefined the FWHM parameter used in CBMA as a cluster density parameter, which depends on the cube root number of studies in the analysis, and is based on the idea that the results of CBMA should be commensurate when performed with different numbers of studies. We have also detailed an algorithm for comparing activation patterns between groups, contrast meta-analysis, using similar methods to our previously described LocalALE algorithm. Statistical testing is performed only at the foci, rather than at each voxel, and our FCDR method of false positive control is employed. Such contrast meta-analyses are only sensitive to very significant localised differences in activation, so we also detail an omnibus test of difference between groups. The omnibus test is sensitive even to subtle diffuse differences between activation patterns, and can provide evidence for a difference where contrast meta-analysis is unrevealing.

By visually inspecting the resulting clusters for several CBMAs, it becomes clear that the FWHM parameter has a major effect on the results. To preserve the cluster characteristics we proposed, by analogy with the density clustering algorithm DBSCAN, that the characteristic volume of each foci 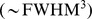 should be adjusted for the number of studies included in the analysis. This method of specifying FWHM completely changes the original meaning that the FWHM represented the spatial uncertainty in the reported foci, for example due to registration error.

Using a typical (10 mm) FWHM, or the FWHM suggested by Eickhoff et. al. [3], results in diminishing or vanishing clusters as the number of studies is reduced, and expanding and merging clusters as the number of studies is increased. This was demonstrated using numerically generated experiments, and by using small selections of studies from real CBMAs. It is clear from the experiments that using a FWHM that is independent of the number of studies could easily lead to the incorrect conclusion that groups with different numbers of studies have different activation patterns; consider the Stroop experiments (figure 4). Figure 7 demonstrates that for increasing study numbers, the cluster sizes increase if the FWHM parameter is not adjusted. This is counter intuitive, as the cluster size and location should be convergent as the experiment size increases. Taking this to its limit of very large numbers of studies, clusters will merge, and study specific foci recruited to them, which is incorrect.

Our proposed method of estimating the FWHM aims to allow overlapping of the high density foci that form clusters, but prevent overlapping of the low density study-specific foci between the clusters. We use an empirical estimate of FWHM based on that which just starts to cause overlapping of foci between different studies under the null hypothesis (figure 5). This is commensurate with the DBSCAN analogy, since at that FWHM the densely packed within-cluster foci overlap, while the between-cluster foci do not. This leads to an estimate of the parameter 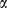 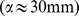 in equation (4).

The experiments depicted in figure 6 are of particular interest because for these we know where the clusters are located, and their approximate size, since this was predefined as part of the experimental procedure [21]. Using 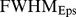 gives results that are reasonable given the original seed ROIs, independent of the number of studies included. Using the FWHM specified in [3], on the other hand, produces rather large clusters for the full dataset. Using these experiments, another estimate of the parameter 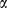 is obtained 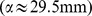.

We have quantitatively analysed the true and false positive rates associated with FWHM estimates using numerical simulation (figures 7 and 8). Three clusters were defined and foci generated to be either true or false cluster members. The results are in keeping with the proposed idea that the FWHM must be adjusted for the number of studies. For very few studies FWHM needs to be increased to allow the foci to overlap and form clusters, albeit with an associated increase in the false cluster rate. Failing to do this could result in missed clusters, as shown by the Stroop experiment. On the other hand, the FWHM must be reduced as the number of studies increases to prevent recruitment of non-cluster foci into the clusters. Indeed the striking observation from figure 8 is that increasing the number of studies in the meta-analysis can be detrimental if the FWHM is fixed due to increased false positives. This is entirely contrary to the aim of meta-analysis, which attempts to reduce uncertainty in estimates.

We have estimated the parameter 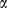 in equation (4) to be 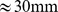, using multiple independent observations; giving the typically used 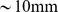 FWHM for an experiment including 30 studies. However, there is some variance on this estimate, and some minor adjustment might be necessary if the clusters are visually fragmented or merging.

Control of false positives in our contrast meta-analysis algorithm is achieved using FCDR; a cluster-level control scheme. Cluster-level schemes have been incorporated into CBMA algorithms previously, and are preferred since they control at the level of interest (clusters) rather than at the voxel-level, but not into algorithms performing contrast of the ALE; indeed no method of control, other than conservative p-value threshold, has previously been described [12].

The contrast meta-analysis and omnibus test for differences between two groups were demonstrated using numerically generated experiments. The first experiment showed the ability of contrast meta-analysis to find local differences, if those differences were very significant. The omnibus test was also able to detect such differences, and with greater sensitivity, but without specifying where the differences are. A second experiment showed that when there are multiple subtle differences between groups, the omnibus test again had more power to detect it than contrast meta-analysis; as expected. Both methods are more sensitive with higher numbers of studies, more significant local differences, or more widespread subtle differences.

Lack of spatial difference in contrast meta-analysis experiments is not a good indicator that there is no difference between the groups being compared because the test is not sensitive to diffuse subtle differences. The omnibus test is more powerful, and should be used where the contrast meta-analysis produces no results. If the omnibus test is not significant, it is more likely that any real difference between groups is small. If it is significant, it can indicate widespread subtle differences between the groups that are undetectable by contrast meta-analysis.

## Conclusions

The FWHM parameter used in ALE coordinate based meta-analysis algorithms is a source of heterogeneity between CBMA results. The meaning of the parameter has been redefined here from being the spatial uncertainty of the reported foci, to a parameter similar to that employed in a density clustering algorithm that works analogously to CBMA. This definition helps reduce the observed heterogeneity. More importantly, fixing the FWHM can paradoxically result in increased false positives as the number of studies increases, while the proposed 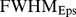 estimate can reduce the false positive rates in the meta-analysis with increasing study numbers.

The many tests used to compare the activation patterns between two groups necessitate a correction for multiple comparisons. We have detailed a contrast meta-analysis algorithm and correction for multiple tests that controls for the proportion of clusters expected under the null hypothesis, the false cluster discovery rate, and takes explicit account of the correlated tests. However, the contrast meta-analysis is not sensitive to diffuse differences between groups. We have therefore detailed an omnibus test that can provide evidence of differences in activation pattern between two groups even when contrast meta-analysis is unrevealing.

## Supporting Information

File S1 **Cluster finding algorithm.**
(DOC)
