# Supplementary material for: Coordinate Based Meta-Analysis of Functional Neuroimaging Data Using Activation Likelihood Estimation; Full Width Half Max and Group Comparisons
Source: PLoS One. 2014 Sep 16;9(9):e106735. doi: 10.1371/journal.pone.0106735 (PMC4165754; doi:10.1371/journal.pone.0106735)
Supplement: File S1 — Cluster finding algorithm. (DOC) [file pone.0106735.s001.doc]

**S1. Cluster finding algorithm**

To assign foci to clusters Dijkstra's shortest path algorithm is utilised. It starts by finding the most significant foci, then assigning the nearby foci to the cluster. Cluster finding is particularly important in CMA since it is the clusters that form the results.

Clusters begin with the most significant foci from the CMA; the central focus. They grow to include further significant foci that overlap each other, and foci that overlap with the significant foci. A cluster is constrained such that the foci p-values should be increasing (but not strictly increasing) away from the central focus. Where clusters merge, such that foci could be assigned to one of several clusters, they are assigned to the closest by the shortest path algorithm.

- Let r*_i_* be the location of the *i*th focus considered for clustering; foci considered are those declared significant by the contrast meta-analysis, and foci that are located <2.8σ from a significant focus
- Let *p_i_* be the p-value for focus r*_i_*
- Let *C_i_* be the cluster focus r*_i_* is assigned to; initialised to zero
- Let δ*_ij_* be the distance separating focus r*_i_* from r*_j_*; δ*_ij_*= δ*_ji_*=|r*_i_ –* r*_j_*|
- Let *d_i_* be the distance from focus *i* from the nearest central focus; initialise to ∞
- Let counter CLUSTER=1

The clustering algorithm uses a heap data structure to make it efficient. In this case foci are entered into the heap, and the data structure sorted such that the focus with the smallest *d_i_* is always at the top, and those foci with the largest *d_i_* always at the bottom. Operations that can be performed on the heap are: **insert**, **remove**, and **update**. These operations are performed such that the heap remains sorted at all times.

1. Find the focus r*_i_* with the smallest *p_i_* and with *C_i_*=0, and set *C_i_*=CLUSTER
2. **Insert** r*_i_* onto the heap, and set *d_i_*=0
3. From all foci currently in the heap, **remove** that with the smallest *d_i_*: r*_k_*
4. For all r*_i_* with δ*_ik_* < 2.8σ AND *p_i_* ≥ *p_k_* AND *d_k_* + δ*_ik_* < *d_i_* do:

Let *d_i_* = *d_k_* + δ*_ik_*

Let *C_i_* = *C_k_*

If r*_i_* not already in the heap, **insert** it now

Otherwise **update** r*_i_* in the heap

1. Repeat from 3 while there are still foci in the heap structure
2. Let CLUSTER = CLUSTER+1
3. Repeat from 1 while there are still foci to assign to clusters
